# P-1320. Antimicrobial susceptibility of S. maltophilia and B. cepacia species complex isolated from patients with pneumonia in United States hospitals (2022-2024)

**DOI:** 10.1093/ofid/ofaf695.1508

**Published:** 2026-01-11

**Authors:** Helio SaderMarisa Winkler, Rodrigo E Mendes, Dmitri Debabov, Mariana Castanheira

**Affiliations:** Element Materials Technology/Jones Microbiology Institute, North Liberty, Iowa; Element Iowa City (JMI Laboratories), North Liberty, IA; Abbvie, Irvine, California; Element, North Liberty, IA

## Abstract

**Background:**

The occurrences of *S. maltophilia* and *B. cepacia* infections, mainly pneumonia, have increased continuously in the last few years. We evaluated the *in vitro* activities of aztreonam-avibactam (ATM-AVI) and comparators against *S. maltophilia* and *B. cepacia* causing pneumonia in United States medical centers.

**Table 1.**
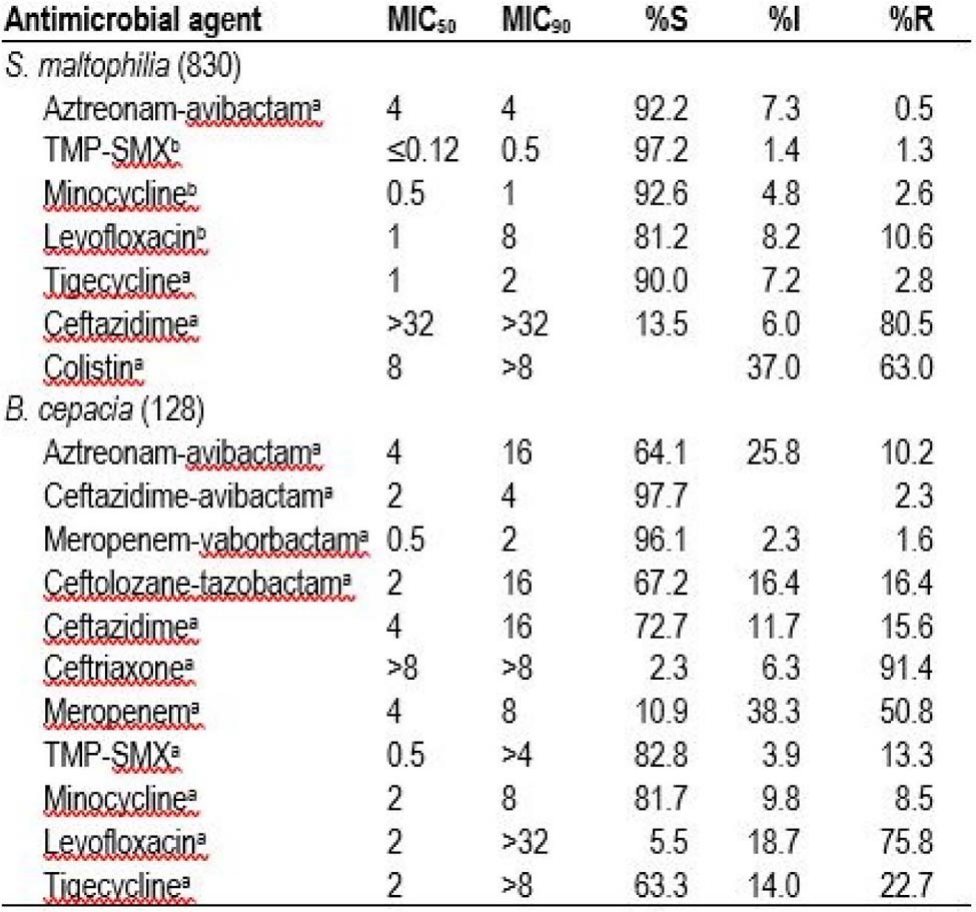
Antimicrobial activity of selected antimicrobials against S. maltophilia and B. cepacia from patients with pneumonia. a CLSI (2025) breakpoints for Enterobacterales were applied for comparison for comparison. b Based on CLSI (2025) breakpoints for S. maltophilia.

**Figure:**
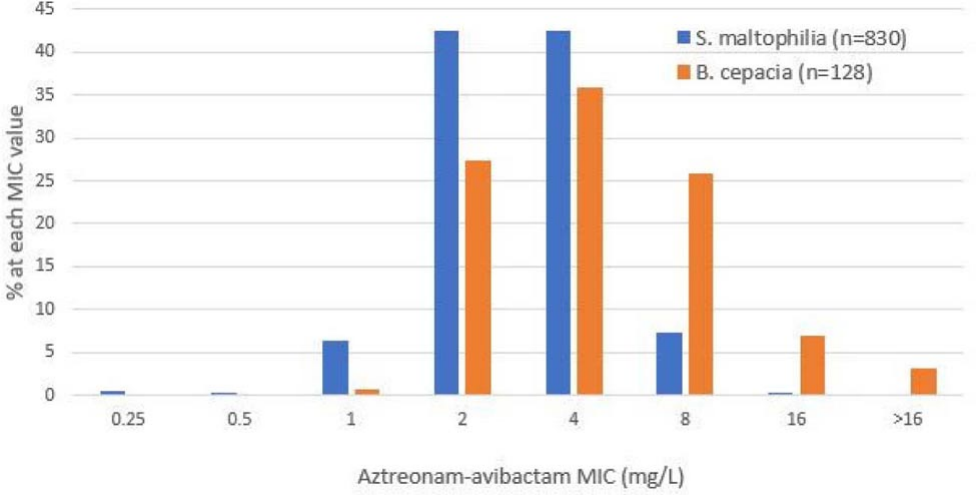
Aztreonam-avibactam MIC distributions for S. maltophilia and B. cepacia from patients with pneumonia

**Methods:**

958 clinical isolates, including 830 *S. maltophilia* and 128 *B. cepacia*, were consecutively collected from patients with pneumonia in 65 United States (US) medical centers in 2022-2024. Isolates were susceptibility tested by CLSI M07 broth microdilution. CLSI breakpoints were applied for *S. maltophilia* when available. Enterobacterales breakpoints were applied to *B. cepacia* and susceptible [S]/resistant breakpoints of ≤ 4/≥ 16 mg/L were applied for ATM-AVI against both organisms for comparison.

**Figure:**
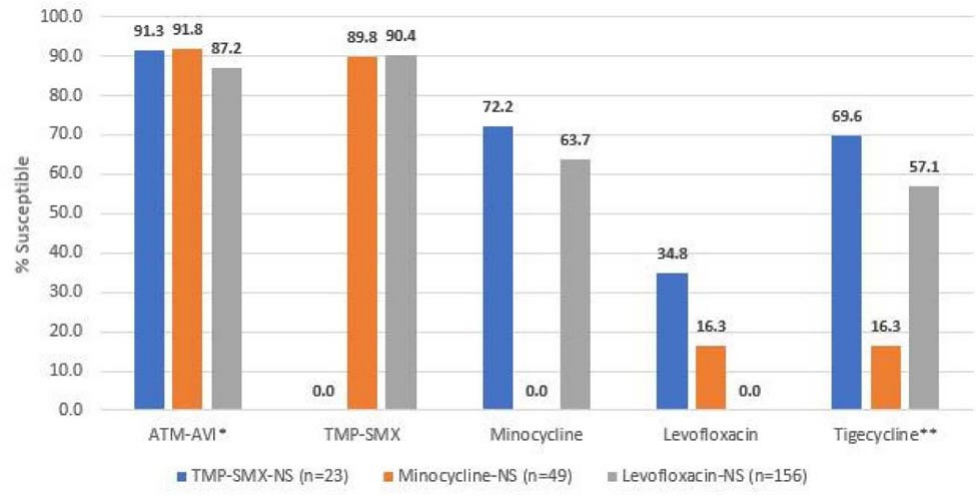
Activity of aztreonam-avibactam and comparators against S. maltophilia resistant subsets. * % inhibited at ≤4 mg/L. ** % inhibited at ≤2 mg/L. Abbreviations: TMP-SMX, trimethoprim-sulfamethoxazole, NS, nonsusceptible.

**Results:**

The most active agents against *S. maltophilia* were trimethoprim-sulfamethoxazole (TMP-SMX; 97.2% S), minocycline (92.6% S) and ATM-AVI (92.2% inhibited at ≤ 4 mg/L; Table 1 and Figure 1). ATM-AVI retained potent activity against isolates non-S (NS) to other agents commonly used to treat *S. maltophilia* infections (Figure 2). Levofloxacin showed moderate activity (81.2%S), tigecycline inhibited 90.0% of isolates at ≤ 2 mg/L (42.5% at ≤0.5 mg/L), and both ceftazidime and colistin exhibited limited activity against S*. maltophilia*. The most active agents against *B. cepacia* were ceftazidime-avibactam (CAZ-AVI; 97.7% inhibited at ≤ 8 mg/L), meropenem-vaborbactam (MEM-VAB; 96.1% inhibited at ≤ 4 mg/L) and TMP-SMX (82.8% inhibited at ≤ 2 mg/L); ATM-AVI inhibited 64.1% at ≤ 4 mg/L and 89.8% at ≤ 8 mg/L. CAZ-AVI and MEM-VAB were active against 91.4% of *B. cepacia* isolates with ceftazidime MIC > 4 mg/L.

**Conclusion:**

ATM-AVI exhibited potent activity and broad coverage against *S. maltophilia* from US hospitals and its activity was not adversely affected by resistance to other agents. The β-lactamase inhibitor combinations CAZ-AVI and MEM-VAB were the most active agents against *B. cepacia* based on CLSI breakpoints published for Enterobacterales. Appropriate assessment of breakpoints for these organisms is urgently needed to provide better guidance of antimicrobial therapy for infections caused by *S. maltophilia* and *B. cepacia*.

**Disclosures:**

Helio Sader, United States Food and Drug Administration: FDA Contract Number: 75F40123C00140 Marisa Winkler, MD, PhD, Basilea: Advisor/Consultant|Basilea: Grant/Research Support|GSK: Advisor/Consultant|GSK: Grant/Research Support|Melinta Therapeutics: Advisor/Consultant|Melinta Therapeutics: Grant/Research Support|Mundipharma: Advisor/Consultant|Mundipharma: Grant/Research Support|Pfizer: Advisor/Consultant|Pfizer: Grant/Research Support|Pulmocide: Advisor/Consultant|Pulmocide: Grant/Research Support Rodrigo E. Mendes, PhD, GSK: Grant/Research Support|Shionogi & Co., Ltd.: Grant/Research Support|United States Food and Drug Administration: FDA Contract Number: 75F40123C00140 Mariana Castanheira, PhD, Melinta Therapeutics: Advisor/Consultant|Melinta Therapeutics: Grant/Research Support

